# Mouse protein coding diversity: What’s left to discover?

**DOI:** 10.1371/journal.pgen.1008446

**Published:** 2019-11-14

**Authors:** Jingtao Lilue, Anu Shivalikanjli, David J. Adams, Thomas M. Keane

**Affiliations:** 1 European Molecular Biology Laboratory, European Bioinformatics Institute, Hinxton, Cambridge, United Kingdom; 2 Instituto Gulbenkian de Ciência, Oeiras, Lisbon, Portugal; 3 Wellcome Sanger Institute, Hinxton, United Kingdom; 4 School of Life Sciences, University of Nottingham, Nottingham, United Kingdom; University College London, UNITED KINGDOM

## Abstract

For over a century, mice have been used to model human disease, leading to many fundamental discoveries about mammalian biology and the development of new therapies. Mouse genetics research has been further catalysed by a plethora of genomic resources developed in the last 20 years, including the genome sequence of C57BL/6J and more recently the first draft reference genomes for 16 additional laboratory strains. Collectively, the comparison of these genomes highlights the extreme diversity that exists at loci associated with the immune system, pathogen response, and key sensory functions, which form the foundation for dissecting phenotypic traits in vivo. We review the current status of the mouse genome across the diversity of the mouse lineage and discuss the value of mice to understanding human disease.

## Author summary

For decades, the laboratory mouse has been widely used to make fundamental discoveries about human biology, model human disease, and develop new treatments. The mouse reference genome is based on the C57BL/6J; however, researchers use a variety of strains to model human disease. Recent genome analysis has identified that the most highly variable regions of the mouse genome are enriched with genes relevant to disease and infection response. In this review, we discuss what is currently known about these regions, why they are important for human disease modelling, and what is known about their ancestral origins.

## Introduction

Although mice and humans have coexisted for many millennia, modern mouse genetics was initiated in the early 20th century [1]. The first genetically homozygous mouse strain was DBA [2], developed to study coat color inheritance and cancer susceptibility. Subsequently, hundreds of genetically defined strains for modelling human diseases and biological processes (e.g., behaviour, carcinogenesis, and immune response against pathogens) were developed. As one of the most important model organisms in biomedical research, the mouse was the second mammalian genome to be sequenced after the human genome [3]. The C57BL/6J reference genome has enabled the creation of detailed molecular maps of mouse diversity [4,5], generation of null alleles, and phenotyping across thousands of genes; and enabled genetic screens at an unprecedented rate [6].

Modern-day laboratory mouse strains are comprised of classical and wild-derived strains. The classical inbred strains have ‘fancy mice’ as their founding ancestors and are largely *Mus musculus domesticus* derived. Other subspecies—*M*. *musculus musculus* and *M*. *musculus castaneus*—contribute approximately 4%–14% to the classical strains [7]. A genome-wide haplotype map of 100 classical mouse strains showed that over 97% of their genome is a mosaic of less than 10 haplotypes [8]. Nevertheless, there are many loci in classical mouse strains, like the major histocompatibility complex (MHC), which have extensive haplotypic diversity [9,10]. Wild-derived inbred mouse strains are recent progenies of wild-caught individuals of *M*. *musculus musculus*, *M*. *musculus castaneus*, and *M*. *spretus* origin and therefore contain many divergent haplotypes not shared with classical inbred strains. Wild-derived strains are increasingly employed as mouse models to study phenotypes—such as resistance against *Orthomyxovirus* [11], virulent *Toxoplasma gondii* strains (CIM, CAST/EiJ, and PWK/PhJ) [12,13], resistance to anticoagulant rodenticides (SPRET/EiJ) [14], and resistance to cerebral *Plasmodium berghei* (WLA/Pas) [15].

## Limitations of a single mouse reference genome

Wild-derived mouse strains have hundreds of thousands of structural differences and novel haplotypes compared to C57BL/6J [4,5]. Most SNP discoveries, if not all, are based on high-density genotyping or short-read sequencing. Paired-end reads are aligned to the C57BL/6J reference genome to identify SNPs, indels, and structure variations (SVs) [16,17]. This means that using the C57BL/6J reference genome to study these strains is blind to many nonreference loci [16]. In these strain-specific diverse regions (SSDRs), next-generation sequencing (NGS) reads are forced to map incorrectly to other paralogous loci in the reference and are often represented as dense regions of heterozygous SNPs (hSNPs) that disrupt the collinearity between the genome of a mouse strain and the reference [13,16]. SSDRs are enriched for genes associated with immunity, sensory, sexual reproduction, and behaviour [16]. In this review, we will introduce the SSDRs among 16 mouse strains and their potential importance in human biomedical research.

**Fig 1:**
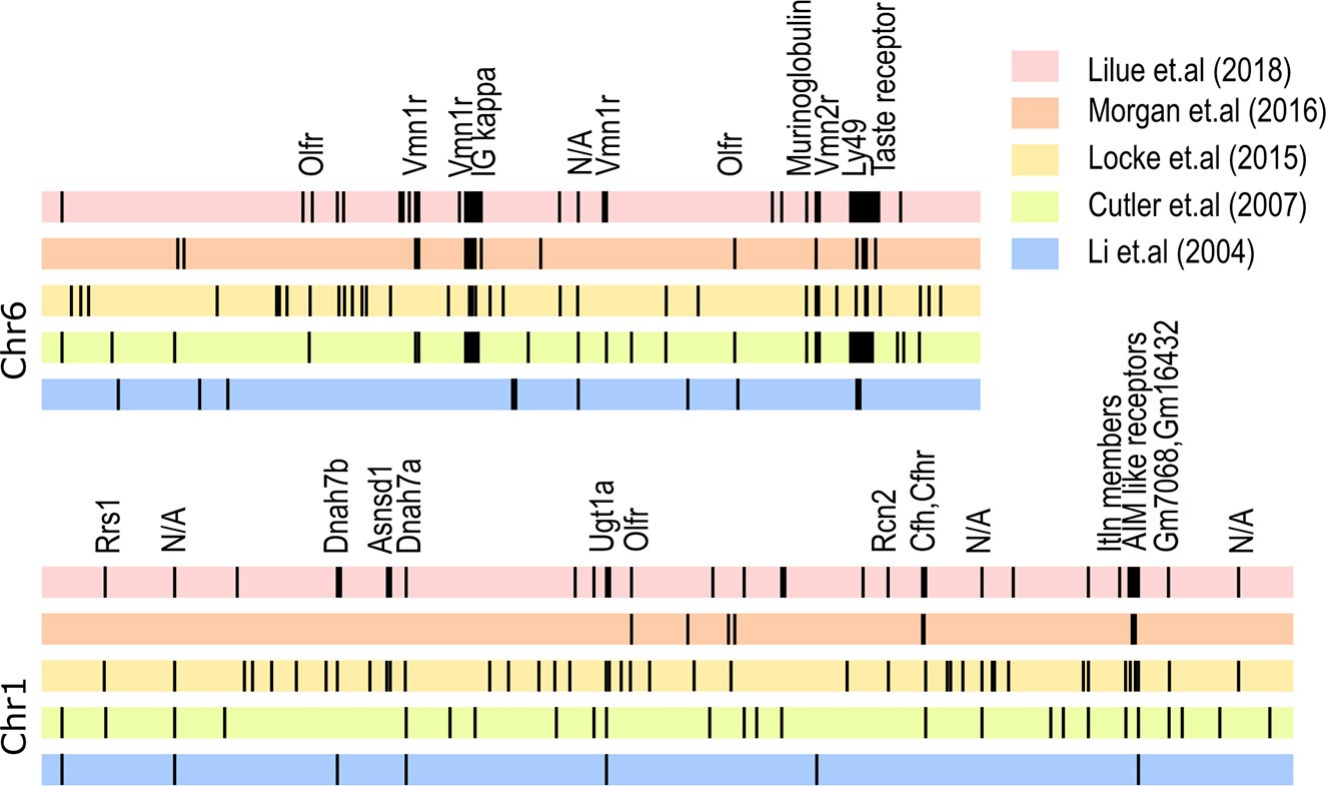
Discovery of SSDRs in the mouse genome. Loci for chromosome 1 and 6 are shown. Li and colleagues [18] defined segmental polymorphisms between 129 and C57BL/6J (blue). Cutler and colleagues [19] cataloged copy number variations in 41 inbred strains (green). Locke and colleagues [20] identified genome regions with high copy number variation calls in 351 different mouse strains and wild-caught mice (yellow). Morgan and colleagues [21] used a combination of wild and inbred mouse strains to define copy number variable regions (orange). Lilue and colleagues [16] used de novo assembly of 16 mouse strains (red). The gene families supported by multiple studies are named above. N/A indicates no protein coding genes in the region. SSDR, strain-specific diversity region.

**Fig 2:**
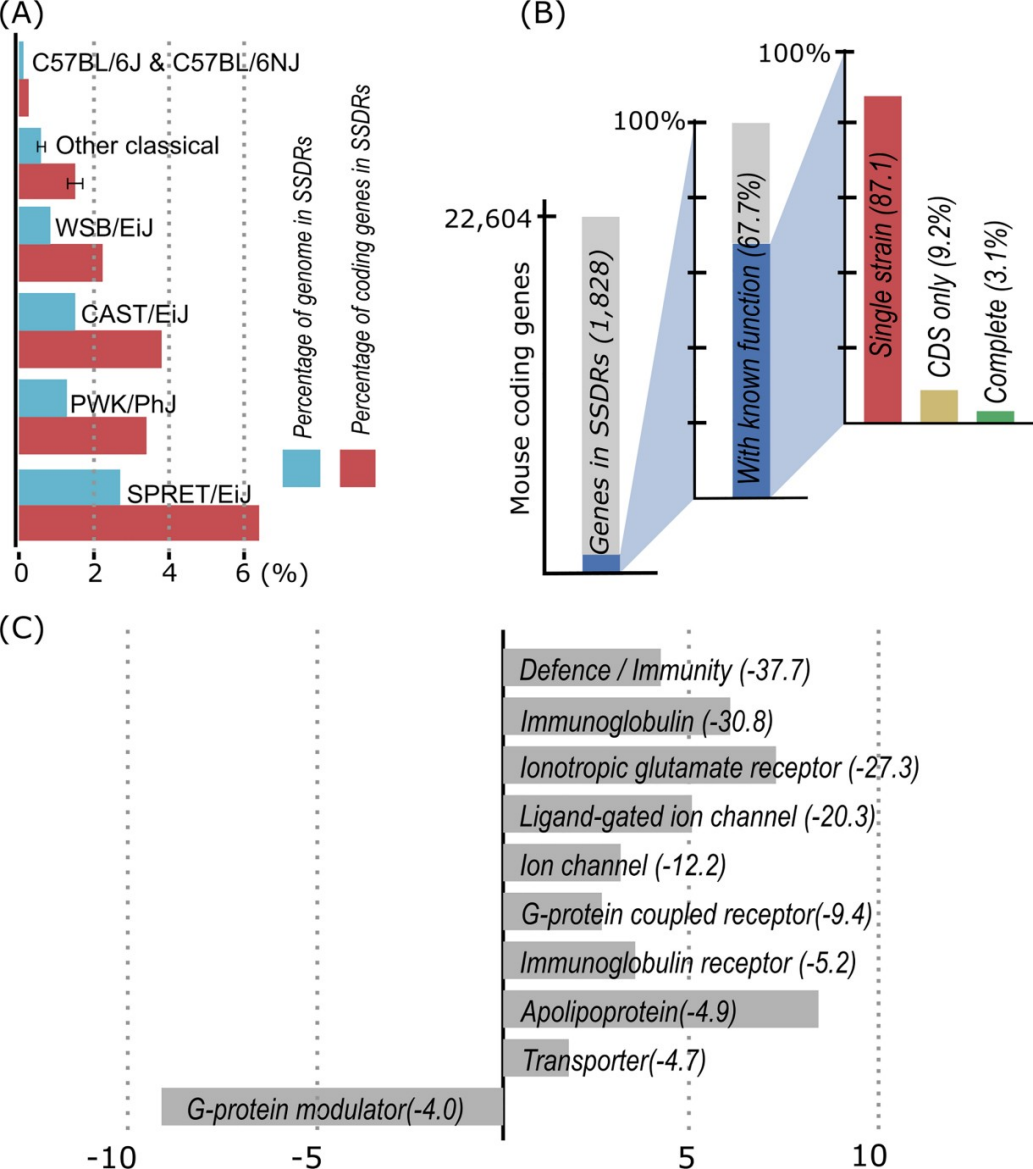
A summary of SSDRs in 16 mouse strains. (A) Proportion of sequence and coding genes in SSDRs for the classical and wild-derived inbred mouse strains. (B) Summary of annotated genes encoded in SSDRs. For gene (families) with known function, only 3.1% have complete sequences (introns and intergenic regions) for multiple mouse strains (green), 9.8% have coding regions from multiple mouse strains (yellow), and all others are based on a single mouse strain (red). (C) Top 10 PANTHER protein classes overrepresented in mouse SSDRs. X-axis indicates times underrepresentation or overrepresentation. Numbers after each protein class indicate corrected FDR(log10 value). CDS, coding sequence; FDR, false discovery rate; SSDR, strain-specific diversity region.

Individual SSDRs associated with phenotypes in mouse inbred strains have long been studied (Fig 1). In 2004, a high resolution whole genome Bacterial Artificial Chromosome (BAC) array analysis reported ‘segmental polymorphisms’ between mouse strains C57BL/6J and 129/Sv [18]. Subsequent work found similar patterns by comparative genomic hybridization analysis and reported 2,094 ‘copy number variations’ (CNVs) in 41 inbred strains [19]. In 2015, an analysis of 351 high-density microarray data for mouse tail samples highlighted 9,634 putative autosomal CNVs affecting 6.87% of the mouse genome [20]. In 2016, Morgan and colleagues performed a genome-wide subsequence diversity test in seven mouse strains and two wild mice samples and reported at least 0.8% of the mouse genome is a ‘genomic revolving door’ with high mutation and recombination rates [21]. In 2018, the first draft de novo assemblies of 16 mouse strains successfully assembled some of these regions, reporting a total of 2,567 SSDRs that encompass 0.5%–2.8% of the mouse genome (Fig 2A and S1 Data), encoding 1,828 coding genes. These genes can be classified into 468 gene families (S1 Table), and 318 (67.7%) have previously been studied in detail. Only 3.1% of gene families have complete sequences (introns and intergenic regions) for multiple mouse strains, 9.8% have coding regions from multiple mouse strains, and most (87.1%) studies draw scientific conclusions based on a single laboratory mouse strain, typically C57BL/6J or a 129 substrain (Fig 2B). SSDRs are enriched for recently transposed long interspersed nuclear elements (LINEs) and long-terminal repeat (LTR) elements, posing a challenge for genome assembly [16] and consequently are often incomplete in the current mouse reference genome.

## Immune-related genes in SSDRs

SSDRs are highly enriched for immunity and infection response related genes (Fig 2C). Examples include MHC, natural killer gene complex [22,23], T-cell receptors [24], and immunoglobulin variable regions [25], which play central roles in non-self-recognition and adaptive immunity. Other loci include oligoadenylate-synthetase 1 (*Oas1*) complex [26], AIM2-like receptors [27], and Schlafen gene family [28] for virus innate immune response; NOD-like receptors—*Nlrp1*—for anthrax lethal toxin resistance [29]; immunity-related GTPases (IRGs) for intracellular pathogen resistance [13]; and α and β defensins for immunomodulatory and antimicrobial function in intestinal crypts [30]. An interesting example is the Intelectin (*Itln*) members encoded on chromosome 1. Intelectin is known to be highly up-regulated in the immune response to parasitic infections, e.g., *Trichinella spiralis*. In the C57BL/6J reference genome, only one *Itln* allele can be found; however, BALB/cJ has two (*Itln1* and *Itlnb*) [31], and strain 129S7 has up to 6 *Itln* alleles (*Itln1* to *Itln6*) [32]. Similarly, the adjacent gene Natural Killer Cell Receptor 2B4 (*Cd244*) shows similar patterns of CNVs in recent de novo assemblies [16].

Recent de novo assemblies have also highlighted many loci with polymorphisms previously unreported in mice. Apolipoprotein L (APOL) members encoded on chromosome 15 show high levels of CNVs in classical and wild-derived mouse strains [16]. There are very few studies in murine ApoL members; however, their orthologues in humans (APOL1) are polymorphic [33]. Some alleles confer resistance to *Trypanosoma brucei brucei* in humans but at the same time lead to chronic kidney disease [33]. *Skint* gene family members, named after ‘skin thickness’ because they regulate epidermal γδ T cells, are associated with chronic wound healing deficiencies in humans [34,35]. A single SNP was reported in mouse strain FVB_Tac_, which causes selective deficiency for epidermal Vγ5^+^Vδ1^+^ T cells [35]. However, the polymorphism of the *Skint* family appears to be much more complex than previously reported among mouse strains. Eosinophil-Associated RNases (*Ears*) encoded on chromosome 14 are orthologues of human eosinophil-derived neurotoxin (EDN) and eosinophil cationic protein (ECP), which are highly charged cytotoxic proteins released from activated eosinophil granules [34]. Mouse *Ears* can promote virus clearance [36] and play a role in the *Schistosoma* resistance [37]. Although evidence of positive selection has been found for *Ears* members in the reference genome, their diversity between inbred strains is poorly documented. At least three haplotypes can be found in classical inbred mouse strains (haplotype1: C57BL/6J, C57BL/6NJ, 129S1, AKR/J, BALB/cJ, A/J, CBA/J, DBA/2J, C3H/HeJ; haplotype2: NZO/HILtJ,LP/J; haplotype3: FVB/NJ, NOD/ShiLtJ), and four wild-derived strains all carry divergent sequences [16]. Signal-regulatory protein beta 1 members (SIRPB1) are cell surface glycoproteins expressed in leukocytes, which positively regulate neutrophil transepithelial migration [38]. A CNV in *SIRPB1* has been reported in humans to be associated with autoimmune thyroid diseases [39] and impulsive-disinhibited personality [40]. In the GRCm38 reference genome, the *Sirpb1* locus remains incomplete [16]. However, the de novo assembly of other inbred mouse strains, especially C57BL/6NJ, has partially improved the reference genome and confirmed significant conservation and high diversity across the strains compared to the C57BL/6J haplotype at the *Sirpb1* locus [16]. Thus, the newly published draft genomes of multiple mouse strains will further facilitate the use of the house mouse for studying human disease.

Both mice and humans carry very large interferon inducible GTPases (GVIN). Their open reading frame is almost 8,000 base pair in length, encoded by a single colossal exon. They are highly expressed in lymph nodes and whole blood in humans [41] and are inducible by both type I and type II interferons (IFNs) in mice [42]. Although the function of GVIN members remains unknown, they are thought to play a role in pathogen immunity [42]. Alpha-1 antitrypsin (AAT) encoded by gene serine protease inhibitor A1 (SERPINA1) is the most abundant antiprotease in humans. It inhibits neutrophil elastase and regulates serine proteases during acute inflammatory responses, especially in the lungs where it protects the fragile alveolar tissues from proteolytic degradation [43]. Human SERPIN family members are highly polymorphic with 1/2,500 newborns in Western Europe carrying the PiZ or PiS allele that causes acute or chronic lung and liver disease [44]. The trade-off of these adverse alleles is still unclear; however, pathogens are reported to manipulate immunity regulation of host as evasion strategies, and SERPINA is a potential target [45]. Among mouse strains, both the *SerpinA* and *SerpinB* gene families are highly polymorphic, and mouse strains with different *Serpin* haplotype may confer a good model for human AAT diversity.

Many more immune-related genes or gene families are encoded in the SSDRs in mice, e.g., IFNs, guanylate-binding proteins (*GBP*), *Ly6* members, orosomucoids (*Orm*), paired-Ig-like receptor (*Pira/Pirb*), interferon-induced proteins with tetratricopeptide repeats (*IFIT*), and CD200 receptors. A summary of these loci can be found in S1 Table.

## Sensory and kin selection

Key to rodent survival is the ability to detect and avoid potentially harmful compounds by smell and taste. The polymorphisms of Tas2r members are believed to match the profiles of bitter chemicals that the mouse population encounter in their diets. Signatures of positive selection have been detected for the human bitter-taste receptor *TAS2R16* [46]. The majority of bitter-taste receptors encoded on mouse chromosome 6 are lineage-specific [47]. Indeed, variations in aversion to chemical substances were observed in BXD mice [48] and between mouse strains C3HeB/FeJ and SWR/J [49]. Both phenotypes were mapped to mouse *Tas2r* loci on chromosome 6 [50].

Olfactory receptors (ORs) are the largest gene superfamily in house mice and most vertebrates [51]. There are 1,296 OR genes distributed in 27 clusters on the Celera mouse genome except chromosome 12 and Y [51]. As one of the most ancient animal senses, olfaction is important to recognise food, identify mates and offspring, and avoid predators or chemical dangers. Polymorphism in ORs in inbred mouse strains is well studied. Multiple OR members from strains 129S1/SvI, 129X1/SvI, 129S6/SvEvTac, A/J, AKR/J, BALB/c, C57BL/6, and DBA/2J were amplified from genomic DNA [51,52] and sequences available [53]. The de novo genome assemblies, especially of wild-derived inbred strains, have greatly boosted the abundance of novel OR genes. Taking strain CAST/EiJ as an example, 1,249 OR candidates have been annotated, 37 of which are not present in the reference mouse genome. In addition, multiple OR pseudogenes in GRCm38 are conserved with CAST/EiJ and vice versa [16]. Strain-specific polymorphisms can be found in 23 OR clusters among 16 mouse strains sequenced. Similar polymorphisms can be found in *Taar7d*, *Taar7e*, *Taar8a*, *Taar8b* and *Taar8c* in wild-derived mouse strains. These members are reported as ORs to recognise ethological odors [54]. The human genome encodes 950 OR genes with high diversity, comparable to the mouse genome [55].

The mouse genome contains many other lineage-specific gene family expansions compared to humans. Many of these genes are associated with reproduction, possibly caused by mating competition and kin selection [56]. One remarkable example is of vomeronasal receptors (VRs) that are mainly expressed in the vomeronasal organ and believed to detect pheromones for sexual recognition. Based on structural differences, VRs are classified into two superfamilies, Vmn1r and Vmn2r, and sum to more than 360 members encoded as clusters on multiple chromosomes in GRCm38 reference genome [57]. The dynamic evolution of VRs and the driving force behind it have been largely discussed in the last decades [58–62]. Wynn and colleagues [57] interrogated around 50% VR genes/alleles from 17 inbred mouse strains and found a significantly higher coding sequence variation with nonrandom distribution in the VRs, especially among three house mouse subspecies and between *M*. *Musculus* and *M*. *Spretus*. These results suggest that VRs may contribute to reproductive isolation between closely related subspecies [57].

As ligands for VRs [63], the major urinary proteins (Mups) are a set of 18–19 kDa communication proteins abundant in mouse urine and other secretions, including lacrimal, parotid, submaxillary, sublingual, preputial, and mammary glands [64,65]. *Mups* may either directly behave as pheromones or bind small molecule pheromones to stabilize them by a slow-release pattern [66,67]. In the house mouse, *Mups* are encoded by a gene-dense cluster on chromosome 4 with at least 19 Mup members per haplotype. Wild mice are reported to express complex ‘barcode’ patterns of Mups, which may provide gender, social dominance, and kinship information to other individuals, facilitating inbreeding avoidance and aiding pup identification. However, wild type individual variation on Mup locus is thought to have been lost during derivation of the classical laboratory strains [68]. It was proposed *Mup* alleles are also highly conserved between individual wild mice [69]. However, previous research based on PCR amplification could not assay novel haplotypes with highly diverse *Mup* members. De novo assemblies of 16 mouse strains have confirmed the sequence diversity in all four wild-derived strains [16].

Another important group of pheromone proteins are exocrine gland secreting peptides (ESPs). They may regulate mouse social behaviours via VR activation. Esp1 is reported to mediate Bruce effect in mice [70], and Esp22 secreted by juvenile mice may inhibit adult male mating behaviour[71]. ESPs are encoded by a gene cluster close to the class I MHC. In the GRCm38 reference genome, 38 *Esp* members are annotated, in which 14 appear to be pseudogenes [72,73]. Although most members in *Esp* family have high sequence diversity between mouse strains, their polymorphisms have not been widely reported. In the human genome, *Mups* and *ESPs* are not present, and all except five *V1R* genes are disrupted by deleterious mutations [74].

## Behaviour and neuron development

Sensory receptors may affect the behaviour of the house mouse directly or indirectly, similar to VRs and ORs [75,76]. The modification of behaviour may also be achieved by regulating the development and connection of neuron cells. For example, protocadherin gamma (*Pcdhg*) members are encoded in a mouse SSDR on chromosome 18. Many *Pcdhg* members show high polymorphism among mouse strains. *Pcdhga* genes are found exclusively in vertebrates and predominantly expressed in the nervous system [77]. They may provide a synaptic address code for neuronal connectivity or a single cell barcode for self-recognition and self-avoidance, and their isoform diversity is necessary for postnatal development of neurons [77].

In humans, male and female specific brain function dimorphisms causing mental impairment have been linked to the X chromosome [78]. This is partially related to X-linked lymphocyte-regulated (*Xlr*) members [79]. *Xlr3b* and *4b* are paternally imprinted in the cortex and other brain regions, which regulate the expression of other genes[79]. *Xlr* genes are encoded on rapidly evolving gene clusters. Among 16 mouse strains, very few SSDRs can be found on the X chromosome, but the two *Xlr* loci give strong signatures of CNVs and novel loci in the wild-derived strains.

One of the most remarkable SSDRs is on chromosome 12 (17–25 mega base pairs [mbp]). This 7 Mbp region encodes hippocalcin-like 1 (*Hpcal1*) and their homologues that belong to the neuronal calcium sensors. Humans have only single copy of *HPCAL1*, which is mainly expressed in retinal photoreceptors, neurons, and neuroendocrine cells [80]. Knockdown of *HPCAL1* in neuroblastoma cells led to impaired neurite outgrowth and inhibited sympathetic neuronal differentiation [81]. In house mice, this gene has been duplicated into 50–100 copies. The mouse *Hpcal1* complex is extremely repetitive containing several recent duplications of hundreds of kilobases. Current draft de novo assemblies do not accurately represent the *Hpcal1* locus in any mouse strain, although it appears that in 12 classical strains at least eight different haplotypes can be observed, and four wild-derived strains contain a further four [16]. To date, the function of *Hpcal1* homologs and the purpose of the rapid expansion of the loci remains unknown. Further candidate genes that potentially have functions in neuron development and regulation include Mas and related G Protein-Coupled Receptors (*Mrgpra*), Angiopoietins (*Ang*), and Neuronal apoptosis inhibitory proteins (Naip) [82–84].

## Sexual reproduction and other biology processes

Sexual reproduction is a complex process from gamete recognition to maternal-fetal interaction. Many genes related to sperm-egg interaction show positive selection and polymorphism, which may reflect the evolutionary pressure from species recognition or inbreeding avoidance [85]. The a disintegrin and metalloprotease (*Adam*) gene family are important sperm surface proteins. Rapid evolution can be found within their sperm-egg adhesion domains [86]. Three *Adam* members are found in a SSDR, namely *Adam20*, *Adam25*, and *Adam26a*. The divergence can be only observed in *M*. *Spretus*, which indicates a potential role in hybridization avoidance [16]. Other sperm specific gene families, however, are polymorphic among classical laboratory mice. Sperm-associated glutamate (E)-rich protein (*Speer*) members are encoded in a gene-dense cluster on chromosome 5. At least three of them are expressed solely in the adult mouse testis. *Speer* homologs are not present in most other mammal species including humans [87], and their function remains unclear. Female specific genes can be also found in the SSDRs. Pregnancy-specific glycoproteins (*Psg*) are members of immunoglobulin superfamily. In humans, PSGs may be the most abundant trophoblastic proteins in maternal blood during pregnancy [88]. Human PSGs play an essential role in the regulation of maternal immunity, by protecting a fetus from immune responses in case of infection, inflammation, and trauma [89]. The polymorphism of *Psg* members in mice is possibly caused by a combination of immune tolerance and host–pathogen coevolution. Four haplotypes of the Psg complex can be found in classical inbred strains, and all wild-derived mouse strains have novel haplotypes.

Many other candidates in the list of mouse strain-specific diversity genes have various function or unknown function (see S1 Table). Variations in keratins and keratin associated proteins (*Krtap*) may affect the hair content characteristics of mouse individuals [90]. Polymorphisms of Hydroxysteroid sulfotransferase enzymes (*Sult*) may reflect challenge from chemical Metabolism. Variation of zinc finger proteins (*Zfp*) are thought to repress transposable elements in an evolutionary arms race [91].

## Conclusions

Isogenic inbred mice have held a unique position as the key mammalian model in evolutionary, genetics, genomics, and biomedical research for over a century. Sequencing and functional studies have documented the extent of genetic polymorphism residing amongst the strains, both shared and unique to each strain. Genetic variation between mouse strains is not evenly distributed across the genome. In most regions, mice are >99.5% identical, but in SSDRs (around 0.5%–2.8% of the mouse genome), the difference is often higher than interspecies diversity between mouse and rat [13]. This scale of diversity cannot be easily represented using the reference genome with SNPs, indels, and SVs. SSDRs are overrepresented with genes associated with immunity, sensory, sexual reproduction, and behavioral phenotypes [16]. The selective pressures driving diversity and CNV includes host–pathogen coevolution (e.g., red queen hypothesis) [92], kin selection [93], mating preference [94], and even selective sweeps due to strong positive selection [95,96]. Many of these genes have direct orthologues in the human genome and are therefore important for understanding health and disease, drug development, and vaccine development. Multiple well-annotated reference genomes will allow researchers to use the appropriate strain for biological rather than historical reasons.

While this review has focused primarily on the limitations of our knowledge of diversity in protein coding regions of the mouse genome, there are other functional elements in which our knowledge is even more limited, e.g., long noncoding RNAs (ncRNAs); piRNAs; and transcription controlling elements such as promoters, enhancers, silencers, and insulators. Multiple reference quality chromosome sequences will provide the foundation for future mapping studies to interrogate these elements. The dramatic drop in second-generation sequencing costs has resulted in genome-wide catalogs of genetic variants for hundreds of mouse strains, but the process of producing a reference quality genome sequence that includes fully resolved novel haplotypes remains costly. Recent advances in third generation sequencing platforms, such as Pacific Biosciences and Oxford Nanopore, can produce mammalian genomes that are an order of magnitude more contiguous[97]. We expect that the representation of many SSDRs in mouse strains will be greatly improved by third generation sequencing platforms.

Human genome-wide association studies (GWAS) have discovered many loci associated with complex disease and traits. Knowledge from model organisms, combined with fine mapping techniques and functional studies, are used to identify causative genes and mechanisms. Mouse SSDR regions are enriched for genes with disease functions with known orthologs in the human genome. The completion of the mouse pan-genome that incorporates all known genetic variants and novel haplotypes will enable the functional characterization of many unresolved quantitative trait loci (QTLs) associated with human disease.

One interesting question is what the origins of these highly diverse haplotypes in the mouse genome are. To date, only a few of these loci have been studied in detail in both inbred and wild mice. Trachtulec and colleagues [98] constructed a haplotype map of the Hst1 region and H2 haplotypes for five mouse subspecies and found that trans-species SNPs were rare, concluding that the haplotypes are unlikely to have arisen by recombination during inbreeding. Lilue and colleagues[13] studied the polymorphic alleles of IRG proteins in inbred laboratory mice that have also been found in European wild mice, suggesting that these alleles arose prior to inbreeding, whilst other more ancient alleles are shared across mouse subspecies. The combination of multiple reference quality genomes for the primary mouse subspecies and availability of larger numbers of sequenced wild mice from ancestral populations will enable a comprehensive analysis of the origins of all SSDRs.

## Supporting information

S1 Table The gene families, publication identifiers, human orthologs, and mouse gene names for the SSDR regions in the mouse genome. SSDR, strain-specific diversity region.
(XLSX)

S1 Data Coordinates on GRCm38 for the SSDR regions per strain (BED format). SSDR, strain-specific diversity region.
(GZ) Click here for additional data file.
